# WHERE DOES EDUCATION COME INTO AGING IN PLACE IN BRAZIL TODAY?

**DOI:** 10.1093/geroni/igae098.1284

**Published:** 2024-12-31

**Authors:** Estela Kohlrausch, Johannes Doll

**Affiliations:** Universidade do Rio Grande do Sul, Porto Alegre, Rio Grande do Sul, Brazil; Federal University of Rio Grande do Sul, Porto Alegre, Rio Grande do Sul, Brazil

## Abstract

Environmental Gerontology has involved a wide range of science, but until now, educational aspects are rarely found. In this paper, we argue that educational theory could give important contributions to the understanding and development of the aging person-environment-relation. There are three fundamental contributions: A) In Environmental Gerontology, there is a lot of thinking about adaptation, stimulation, and orientation processes in dealing with changes in the environment or changes in the person during aging process. These processes can be understood as learning processes, and Education Science could contribute by appointing conditions and obstacles for those processes, as for example Peter Jarvis’ existential learning theory for adults. B) Educational theory points out that learning processes happens in social and cultural contexts by social interaction, as Paulo Freire stated: Nobody educates anyone, nobody educates themselves, men (and women) educate each other, mediated by the world. C) Educational processes are intentional, by respecting the other in a dialogical relation. This might be an interesting form for gerontological intervention and is in line with actual discussion about participatory approaches in aging research. Within the major changes in society such as globalization, urbanization, digitalization and climate change, a critical educational perspective, as for example Paulo Freire’s liberation pedagogy can help to understand environment in a broader context, not only to guarantee safety and comfort for older people, but by “reading the world” (Paulo Freire) people can start to get aware of one´s own role within the larger context of community, society and the human world.

